# Age-Related Serum Biochemical Reference Intervals Established for Unweaned Calves and Piglets in the Post-weaning Period

**DOI:** 10.3389/fvets.2019.00123

**Published:** 2019-04-24

**Authors:** Kuai Yu, Francesca Canalias, David Solà-Oriol, Laura Arroyo, Raquel Pato, Yolanda Saco, Marta Terré, Anna Bassols

**Affiliations:** ^1^Departament de Bioquímica i Biologia Molecular, Facultat de Veterinària, Universitat Autònoma de Barcelona, Barcelona, Spain; ^2^Departament de Bioquímica i Biologia Molecular, Facultat de Medicina, Universitat Autònoma de Barcelona, Barcelona, Spain; ^3^Animal Nutrition and Welfare Service, Animal and Food Science Department, Facultat de Veterinària, Universitat Autònoma de Barcelona, Barcelona, Spain; ^4^Servei de Bioquímica Clínica Veterinària, Facultat de Veterinària, Universitat Autònoma de Barcelona, Barcelona, Spain; ^5^Departament de Producció de Remugants, Institut de Recerca i Tecnologia Agroalimentàries, Barcelona, Spain

**Keywords:** serum clinical biochemistry, reference interval, non-parametric, unweaned calf, weaning piglet, age

## Abstract

The purpose of the present study is to establish the influence of age on serum biochemistry reference intervals (RIs) for unweaned calves and recently-weaned piglets using large number of animals sampled at different ages from populations under different season trials. Specifically, milk replacer (MR)-fed calves from April–July 2017 (*n* = 60); from December 2016–March 2017 (*n* = 76) and from April–August 2018 (*n* = 57) and one group of healthy weaned piglets (*n* = 72) were subjected to the study. Serum enzymes and metabolites of calves at age of 24 h (24 h after colostrum intake), 2, 5, and 7 weeks from merged trials and piglets at 0, 7, and 14 days post-weaning (at 21, 28, and 35 days of age) were studied. The main variable is age whereas no major trial- or sex-biased differences were noticed. In calves, ALT, AST, GGT, GPx, SOD, NEFAs, triglycerides, glucose, creatinine, total protein, and urea were greatly elevated (*p* < 0.001) at 24 h compared with other ages; glucose, creatinine, total protein, and urea constantly decreased through the age; cholesterol's lowest level (*p* < 0.001) was found in 24 h compared with other ages and the levels of haptoglobin remained unchanged (*p* > 0.1) during the study. In comparison with the adult RIs, creatinine from 24 h, NEFAs from 2 w, GGT from 5 w, and urea from 7 w are fully comparable with RIs or lie within RIs determined for adult. In piglets, no changes were noticed on glucose (*p* > 0.1) and haptoglobin (*p* > 0.1) and there were no major changes on hepatic enzymes (ALT, AST, and GGT), total protein, creatinine and urea even though several statistical differences were noticed on 7 days post-weaning. Cholesterol, triglycerides, NEFAs, cortisol and PigMAP were found increased (*p* < 0.05) while TNF-alpha was found less concentrated (*p* < 0.001) at 0 days post-weaning compared with other times. Moreover, the RIs of creatinine and GGT are fully comparable with RIs or lie within RIs determined for adult. In conclusion, clinical biochemistry analytes RIs were established for unweaned calves and recently-weaned piglets and among them some can vary at different ages.

## Introduction

Serum biochemical reference intervals (RIs) play an important role in assessing animal health. However, for farm animals, the majority of RIs are set for adult individuals. Similar to human medicine, where pediatric RIs are readily available, there is a necessity of robust and specific RIs for young animals in veterinary science, since their physiology is different from adults and it may be markedly affected by milk-feeding and weaning. The use of adult RIs intervals may be inadequate since young animals may metabolize in a different way. Furthermore, young animals must be closely monitored since they are more vulnerable to diseases, and their diets may have a bigger impact on the health condition in comparison with the adult. Thus, many of the RIs publications in veterinary science established for adults (1, 2) may be misleading (3) and the establishment of RIs in young animals is needed to adequately discriminate between healthy and diseased individuals.

In newborn calves, their growth involves several morphological, metabolic and physiological changes (4) and these changes may be reflected by metabolites and enzymes' levels in blood (5, 6). Calves undergo metabolic and digestive tract physical changes during the weaning process (in the dairy industry it occurs between 6 and 9 weeks of age) (7). At birth, most of the nutrients (glucose, amino acids, and long-chain fatty acids) from milk are digested in the abomasum and absorbed in the small intestine. Thus, young calves are non-ruminant. As calf starts to consume solid feeds (like concentrate feeds), the rumen starts to develop. At weaning, the rumen is mostly developed and it supports part of the nutrient absorption from solid feed fermentation. This different nutrient supply at weaning also entails a shift in the hepatic function from a glycolytic to glucogenic liver altering metabolites and enzymes levels in blood.

In swine industry, an “early weaning” is commonly used with the objective of reducing and improving the sow productive cycle. Normally weaning occurs abruptly at 21–28 days of age and the piglet needs to be adapted to eat a novel food (usually solid diet) after the separation from the sow. In contrast to the natural weaning which gradually process until 17 weeks of age (8), early weaning may become stressful for piglets. The immediate post-weaning period is critical for the piglet health since at that moment they are very susceptible to gastrointestinal problems and infections.

In both species, the early stage age involves several biochemical and metabolic changes that may be fundamentally different from the adult ones. Moreover, since young animals are more vulnerable to certain pathogens, basic health laboratory routine check based on serum biochemical analytes is essential.

In the past 20 years, neonatal clinical biochemistry gradually has been established and the biochemical RIs on unweaned cattle calves of different breeds and ages up to 3 months of old (3, 9–11) and for piglets younger than 1 month of age (12, 13) have been published. In all these studies, several noticeable biochemical changes along the age were reported. Nevertheless, these studies have two main drawbacks. First, RIs have been calculated from small populations (<35), and this does not meet the requirement of non-parametrical RI. As it is recommended in Clinical and Laboratory Standards Institute (CLSI) Guideline, a non-parametric method is recommended due to its simplicity and reliability (14), thus a sufficient population for RI establishment could be more trustworthy. Second, many RIs are calculated from individual experiments, and since factors like diet, geography, season, sex, and breed may affect the RI, thus the obtained value may not applicable in other conditions.

In this study, the first objective is to determine clinical biochemistry values from a large set of healthy unweaned calf populations from 3 different nutritional programs and breeding seasons at 24 h, 2 weeks, 5 weeks, and 7 weeks of age; the second objective is to determine healthy recent-weaned piglets' clinical biochemistry values from both sexes at 0 days, 7 days, and 14 days post-weaning and the third objective is to evaluate all the values and establish RIs. The purpose is to obtain RIs useful for the veterinary practitioner and for research.

## Materials and Methods

### Calf Housing and Diet Management

Male Holstein calves were managed according to the recommendations of the Animal Care Committee of Institut de Recerca i Tecnologia Agroalimentàries (IRTA) under the approval research protocol FUE-2017-00587321, authorization code 9733. Holstein male calves were obtained from Granja Murucuc (Gurb, Barcelona, Spain) and housed individually at IRTA in Torre Marimon (Barcelona, Spain). Plastic ear tag identification with the animal's number was used. In all cases, animals were under veterinary control and no health problems were observed during the experimental period. The total number of individuals included in the study was *n* = 193. Animals belonged to three trials.

In Trial 1 (T1), 60 new-born calves (age = 0 h) were fed with colostrum within the first 6 h after birth. At 24 h of age, blood samples were taken. Then, at age of 3 ± 1.3 days old, animals were fed with a MR and blood samples were collected at 2 and 5 weeks of age 4 h after the morning feeding. The T1 was carried out between April and July 2017.

In Trial 2 (T2), 76 calves (average age = 3 ± 1.7 d) were fed with MR and blood samples were collected at 2, 5, and 7 weeks of age 4 h after the morning feeding. The T2 started in December 2016 and ended in March 2017.

In Trial 3 (T3), 57 calves (average age = 5 ± 2.2 d) were fed with MR and blood samples were taken at 2, 5, and 7 weeks of age 4 h after the morning feeding. The T3 was carried out between April and August 2018.

All animals followed the same MR feeding program that consisted on feeding 2-L of MR (Nukamel, Weert, The Netherlands) twice a day containing at 12.5% concentration of MR powder (500 g/d) for the first 4 days, subsequently MR was increased to 5 L/d of MR at 12.5% concentration of MR powder (625 g/d) for the next 3 days, then MR was increased to 6 L/d of MR at 12.5% concentration of MR powder (750g/d) for the next 7 days and then 2 meals of 3 L at 15% concentration (900 g/d) until 49 d of age. Then, MR was limited to a single offer of 3 L also at 15% (450 g/d) until end of the study. Animals also had access to water and chopped barley straw *ad libitum*. Chemical composition and ingredient of the MRs and the starter intake information are shown in Tables S1, S2.

### Piglet Housing and Sampling

A total of 336 commercial crossing piglets ([Large White x Landrace] x Pietrain), male and female 21-d old piglets of 4–7 kg of body weight (BW) were weaned. The animals were moved from the farrowing to the nursery unit (within the same commercial farm without transport) in the morning on the day of weaning. Plastic ear tag identification with the animal's number was used. In the nursery room, pigs were then allocated in 24 pens (14 piglets/pen). Each pen has had a commercial non-lidded hopper and a nipple waterer to ensure *ad libitum* feeding and free water access. The experimental procedure has been approved by ethical committee (CEEAH) of Universitat Autònoma de Barcelona (Registration n°:1406 of the Departament de Medi Ambient i Habitatge of the Generalitat de Catalunya).

At weaning, animals were offered the same basal pre-starter diets following the same specification. The pre-starter and starter diets were fed *ad libitum* for fourteen consecutive days (P1) and 21 consecutive days (P2), respectively. A basal diet was formulated to contain 2,470 kcal NE/kg; 20.5% CP/kg and 1.35% Dig Lys for P1 and 2,400 kcal NE/kg; 18.1% CP/kg and 1.20% Dig Lys for P2. Diets were formulated to meet the requirements for growth of newly weaned piglets (15). No antibiotics and ZnO were included in the basal diets.

Blood samples from 3 piglets per pen (a total of 72 animals) were collected avoiding cross-contamination between pens at 8 am at week 3 of age (day 0 after weaning), week 4 (day 7 after weaning), and week 5 (day 14 after weaning). Piglets were individually weighted before weaning and distributed for a balanced body weight within each pen. Piglets were sorted by descending BW within each pen and the one in the middle (median), the ones immediately above and below the median were picked out to be sampled. The same animals per pen were sampled on weeks 4 and 5, respectively.

### Sample Collection and Analysis

Blood samples obtained from jugular venipuncture were collected in 10 ml Vacutainer tubes without anticoagulant. Sera were obtained by centrifugation at 1,500 g for 10 min and transferred to new tubes to be stored in aliquots at −80°C until further analysis. At the day of analysis, aliquots were thawed. Measurement of serum clinical biochemistry analytes was performed on the Olympus AU400 analyser except cortisol and TNF-alpha. The protocols for the use of Olympus System Reagents (OSRs) and other commercial reagents were in reference of previous studies (16, 17). Quality control protocols were based on the daily quantification of two control sera of low and high concentration (Control serum I and Control serum II, Beckman Coulter). For cortisol and TNF-alpha in piglets, two commercial ELISA kits were used: Cortisol ELISA Kit (DRG, Marburg, Germany) and Porcine TNF-alpha Quantikine ELISA Kit (R&D Systems, Abingdon, UK). The analytes, methods, reagents, and coefficients of variation (CV%) are shown in Table S3.

### RI Establishment and Statistics

The RI establishment was in accordance with American Society for Veterinary Clinical Pathology (ASVCP) guidelines (18). These guidelines mirror the CLSI recommendations. All RIs were generated by Reference Value Advisor (19), an add-in program in Excel (Office 365, Microsoft, Redmond, WA, USA). For population amounts over 120, non-parametric RIs were directly used since this method doesn't assume any specific shape for data distribution. For populations <120 (24 h of age for calves and piglet samples), non-parametric RIs were not able to be computed. The criteria of outlier removal are following: (a) For interquartile range (IQR) = Q_3_(third quartiles)—Q_1_(first quartiles), values that exceed interquartile fence set at Q_1_−3^*^IQR and Q_3_+3^*^IQR are considered outliers; (b) Without removing certain outliers, if Reference Value Advisor computes result with good distribution and/or symmetricity, then the outliers are retained; (c) If no normal distribution and/or symmetricity are observed even outliers are removed and data are power transformed, such population is calculated with Cook's distance and Cook's outliers are detected and deleted. After the removal of outliers, populations were recalculated for RIs. Once the RIs were established, one-way analysis of variance (ANOVA) and Tukey's HSD with Bonferroni correction test, boxplots with difference annotations and distribution histograms were made by “R” (20) under RStudio environment (21) using the combinations of “multcompView” (22), “nortest” (23), “ggplot2” (24), “gridExtra” (25), and “ggpubr” (26) packages and values that exceed interquartile fence set at Q_1_−3^*^IQR and Q_3_+3^*^IQR were considered outliers.

## Results

For calves and piglets, the frequency of distribution histograms of all analytes from all the individuals (both trial-wise and merged-data-wise) were visualized before the RIs were established (shown in Figures S1, S2). The distribution from the different sets of animals overlapped and differences between groups were not significant, indicating that no year season, experimental or sex-biased distributions were found and in consequence, all the individuals of the same age were included in the same reference population. There were only two exceptions identified in calves: AST and cholesterol at week 2, where T3's population displayed a non-overlapping distribution curve.

For calves, the methods of RI calculations of 24 h was parametric since population consisted in 30 individuals and from 2 w non-parametric method was used as the populations are over 120.

For piglets, since the population size didn't reach 60, parametric method was used for all ages and analytes except one non-parametric method was suggested using by the software on AST at 7 d due to unfixable normality of distribution.

**Figure 1:**
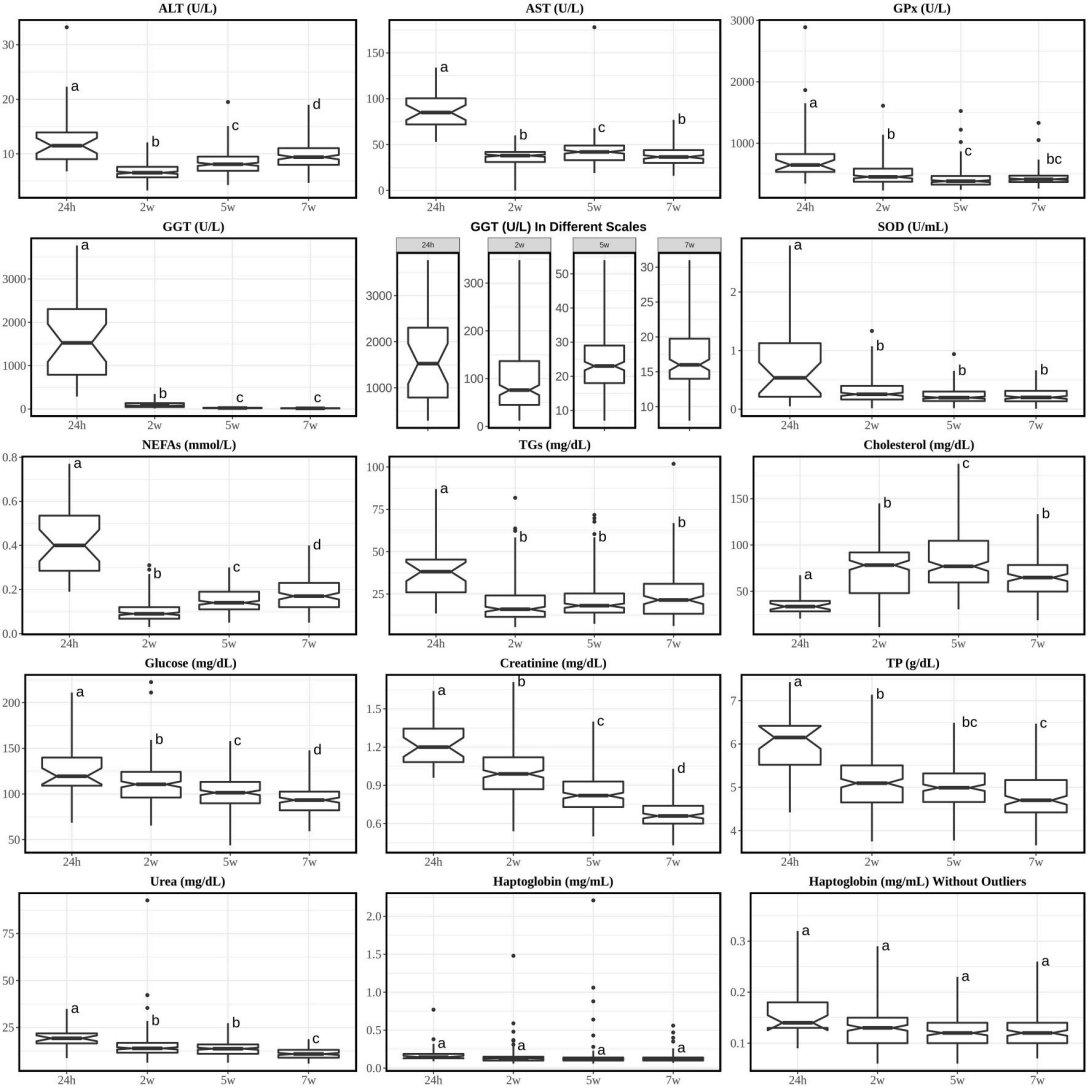
Boxplots of analytes with Tukey's HSD *posthoc* with Bonferroni correction from calves' sera at 24 h, 2 w, 5 w, and 7 w of age. Annotations over the boxes with different superscripts are significantly different (*p* < 0.05). Interquartile fence was set between Q1-3*IQR and Q3+3*IQR. 24 h, calves at 24 hours of age; 2 w, calves at 2 weeks of age; 5 w, calves at 5 weeks of age; 7 w, calves at 7 weeks of age. Analytes were alphabetically plotted according to the orders of discussion.

**Figure 2:**
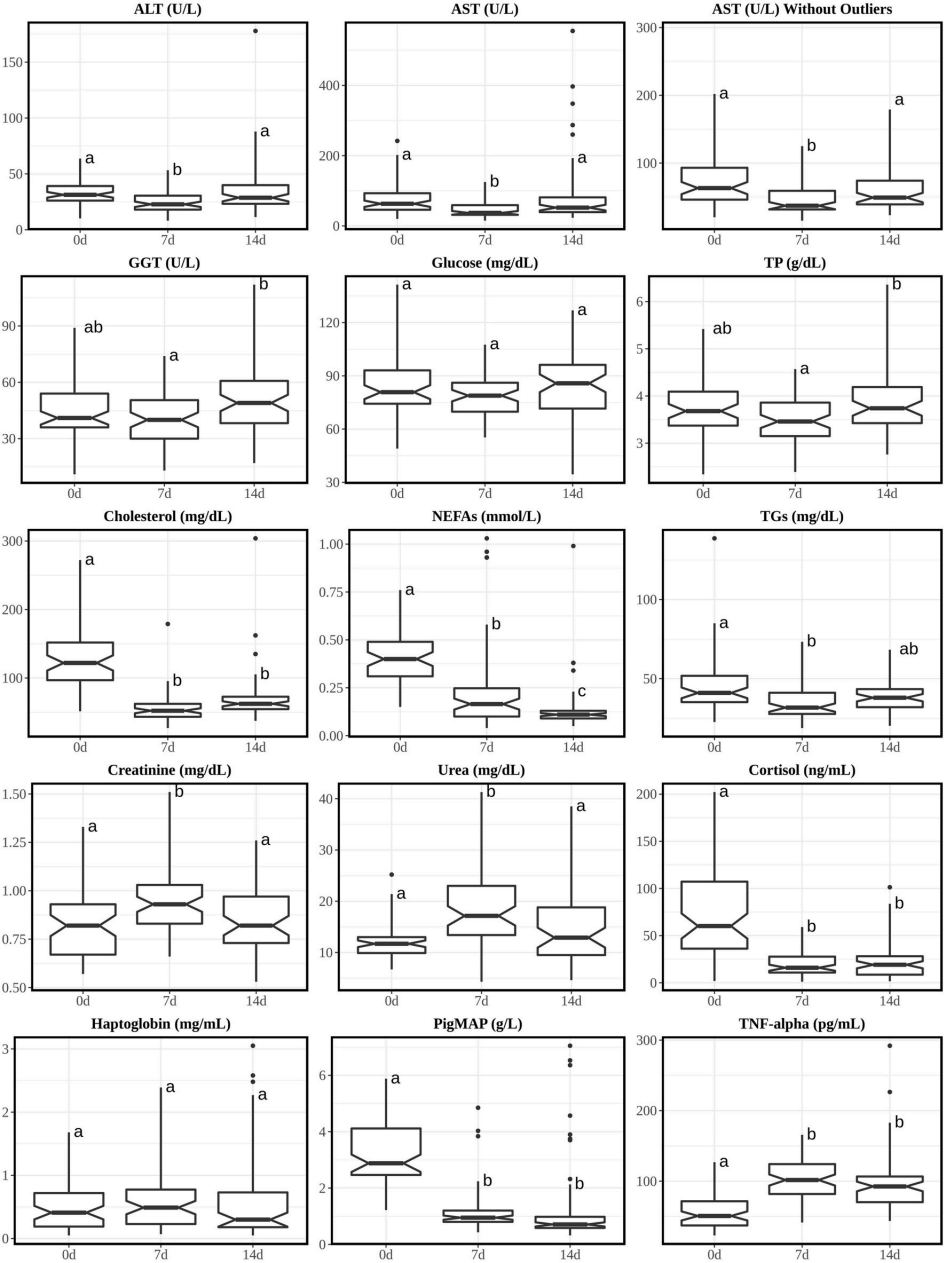
Boxplots of analytes with Tukey's HSD *posthoc* with Bonferroni correction from piglets' sera at 0, 7, and 14 days post-weaning (age: 21, 28, and 35 days, respectively). Annotations over the boxes with different superscripts are significantly different (*p* < 0.05). Interquartile fence was set between Q1-3*IQR and Q3+3*IQR. 0d, piglets at the 0 day of weaning (right after weaning); 7d, piglets at the 7th day of weaning; 14d, piglets at the 14th day of weaning. Analytes were alphabetically plotted according to the orders of discussion.

A summary of the results of both species at different ages are shown in Tables 1, 2, respectively. Figures 1, 2 show the pattern of age-related differences for all parameters. The *post-hoc* multi-comparison results are shown in Tables S4, S5. Missing and undetectable samples were discarded in this study.

**Table 1:** Summary of statistical values for clinical biochemistry analytes from calves of 24 h (24 h after colostrum taking), 2, 5, and 7 weeks of age.

	**Analyte**	**ALT**	**AST**	**Cholesterol**	**Creatinine**	**GGT**	**Glucose**	**GPx**	**Haptoglobin**	**NEFAs**	**TGs**	**TP**	**SOD**	**Urea**
	**Unit**	**U/L**	**U/L**	**mg/dL**	**mg/dL**	**U/L**	**mg/dL**	**U/L**	**mg/mL**	**mmol/L**	**mg/dL**	**g/dL**	**U/mL**	**mg/dL**
24 h	Mean	12.1	87.5	36.1	1.22	1459.8	125.8	737.1	0.16	0.41	39.1	6.02	0.90	19.4
	Median	10.8	85.0	33.6	1.20	1303.0	119.4	632.0	0.14	0.40	38.2	6.15	0.53	19.3
	SD	4.4	20.4	11.1	0.17	831.3	28.5	347.4	0.05	0.15	19.7	0.71	0.89	5.6
	Minimum	6.8	53.0	20.6	0.96	288.0	68.5	343.1	0.09	0.19	13.5	4.42	0.05	8.7
	Maximum	22.3	134.0	67.4	1.64	3170.0	211.1	1650.4	0.32	0.77	87.0	7.43	2.79	34.9
	N	29	30	30	30	28	30	28	28	30	30	30	30	30
	Method	Trans. Std.	Trans. Std.	Trans. Std.	Trans. Std.	Trans. Std.	Untrans. Std.	Trans. Std.	Trans. Std.	Untrans. Std.	Trans. Std.	Trans. Std.	Trans. Std.	Trans. Std.
2 w	Mean	6.8	36.6	71.5	1.00	100.8	110.6	505.0	0.14	0.10	19.6	5.11	0.31	15.2
	Median	6.6	38.0	78.3	0.99	76.0	110.6	452.4	0.13	0.09	16.0	5.10	0.25	13.9
	SD	1.7	9.3	29.3	0.20	70.6	22.1	194.0	0.12	0.06	12.1	0.63	0.21	7.9
	Minimum	3.3	0.0	11.5	0.54	12.0	65.4	231.6	0.06	0.03	5.5	3.75	0.02	6.3
	Maximum	12.1	60.0	145.0	1.71	348.0	222.5	1610.7	1.48	0.31	81.8	7.14	1.33	92.7
	N	188	188	188	188	188	188	186	190	188	188	188	185	188
	Method	NP	NP	NP	NP	NP	NP	NP	NP	NP	NP	NP	NP	NP
5 w	Mean	8.3	42.2	82.1	0.83	24.5	101.8	421.1	0.14	0.15	21.9	4.99	0.23	13.9
	Median	8.1	42.0	77.0	0.82	23.0	101.4	383.0	0.12	0.14	18.1	4.99	0.20	13.7
	SD	2.1	15.0	29.4	0.16	9.5	18.4	157.6	0.18	0.06	12.0	0.50	0.14	3.9
	Minimum	4.3	19.0	30.6	0.50	7.0	43.8	242.2	0.06	0.05	7.4	3.77	0.02	6.3
	Maximum	19.5	178.0	187.7	1.40	54.0	157.9	1525.3	2.21	0.30	71.7	6.49	0.94	27.3
	N	193	193	193	193	193	193	192	193	193	193	193	191	193
	Method	NP	NP	NP	NP	NP	NP	NP	NP	NP	NP	NP	NP	NP
7 w	Mean	9.5	37.5	65.5	0.67	16.8	93.0	437.1	0.13	0.18	24.1	4.80	0.23	11.2
	Median	9.4	36.5	64.9	0.66	16.0	93.3	413.2	0.12	0.17	21.5	4.70	0.20	10.9
	SD	2.4	10.8	23.0	0.12	4.4	15.3	128.0	0.06	0.07	14.3	0.54	0.13	2.9
	Minimum	4.7	16.0	18.8	0.43	8.0	59.2	262.4	0.07	0.05	6.1	3.66	0.01	5.8
	Maximum	19.0	77.0	133.4	1.03	31.0	147.8	1330.8	0.56	0.40	101.9	6.47	0.66	18.8
	N	134	134	134	134	134	134	133	134	134	134	134	130	134
	Method	NP	NP	NP	NP	NP	NP	NP	NP	NP	NP	NP	NP	NP

*SD, Standard Deviation; N, Number of animals; ALT, Alanine Aminotransferase; AST, Aspartate Aminotransferase; GGT, Gamma-Glutamyl Transferase; GPx, Glutathione Peroxidase; NEFAs, Non-Esterified Fatty Acids; TGs, Triglycerides; TP, Total Proteins; SOD, Superoxide Dismutase*.

*Methods: NP, Non-parametric; Trans. Std., Transformed data using standard method; Untrans. Std., Untransformed data using standard method*.

**Table 2:** Summary of statistical values for clinical biochemistry analytes from piglets at 0, 7, and 14 days post-weaning (at 21, 28, and 35 days of age).

	**Analyte**	**ALT**	**AST**	**Cholesterol**	**Cortisol**	**Creatinine**	**GGT**	**Glucose**	**Haptoglobin**	**NEFAs**	**PigMAP**	**TGs**	**TNF-alpha**	**TP**	**Urea**
	**Unit**	**U/L**	**U/L**	**mg/dL**	**ng/L**	**mg/dL**	**U/L**	**mg/dL**	**mg/mL**	**mmol/L**	**g/L**	**mg/dL**	**pg/mL**	**g/dL**	**mg/dL**
0 d	Mean	32.8	78.4	125.5	74.9	0.80	46.0	84.9	0.50	0.40	3.26	43.2	54.3	3.78	12.1
	Median	31.3	63.0	122.0	60.2	0.79	41.0	80.8	0.41	0.40	2.88	40.9	48.8	3.68	11.7
	SD	9.4	47.5	41.8	50.1	0.15	16.5	17.8	0.39	0.14	1.22	13.1	22.3	0.64	3.6
	Minimum	10.2	20.0	51.4	2.0	0.57	11	49.0	0.05	0.15	1.22	22.6	23.1	2.34	6.7
	Maximum	63.7	242.0	272.2	202.2	1.16	89	141.4	1.68	0.76	5.88	85.0	110.6	5.42	25.2
	N	66	68	61	71	54	65	57	61	61	71	59	67	58	57
	Method	Trans. Std.	Trans. Std.	Trans. Std.	Trans. Std.	Untrans. Rob.	Trans. Std.	Untrans. Rob.	Trans. Std.	Trans. Std.	Trans. Std.	Trans. Std.	Trans. Std.	Trans. Std.	Trans. Std.
7 d	Mean	24.8	45.8	53.1	19.0	0.94	40.9	79.1	0.62	0.18	0.99	35.5	103.2	3.49	18.2
	Median	22.8	37.0	52.0	15.6	0.93	40.0	78.9	0.49	0.15	0.94	31.7	101.7	3.46	17.2
	SD	8.8	22.3	14.3	12.4	0.16	13.9	12.4	0.52	0.11	0.31	11.2	27.7	0.48	6.9
	Minimum	8.2	15.0	27.0	1.1	0.66	13	55.3	0.07	0.04	0.43	18.8	41.4	2.39	4.3
	Maximum	53.3	125.0	95.5	52.5	1.51	74	107.6	2.39	0.58	2.24	73.3	165.7	4.57	41.3
	N	67	67	65	68	65	67	64	66	62	69	65	72	65	64
	Method	Trans. Std.	NP^*^	Trans. Std.	Trans. Std.	Trans. Std.	Trans. Std.	Trans. Std.	Trans. Std.	Trans. Std.	Trans. Std.	Trans. Std.	Trans. Std.	Trans. Std.	Trans. Std.
14 d	Mean	35.5	65.6	69.7	21.2	0.85	50.9	83.0	0.57	0.11	0.74	38.8	98.1	3.84	14.7
	Median	28.7	48.0	62.4	18.9	0.82	49.0	85.8	0.30	0.10	0.70	38.0	92.5	3.74	12.9
	SD	23.6	45.7	36.9	16.7	0.17	17.4	16.5	0.66	0.03	0.28	10.0	42.8	0.69	7.5
	Minimum	11.4	23.0	37.5	1.6	0.53	17	34.5	0.05	0.05	0.32	20.2	43.6	2.76	4.6
	Maximum	177.9	260.0	303.9	83.8	1.26	112	126.9	3.05	0.21	2.13	68.3	292.0	6.36	38.5
	N	64	62	62	65	57	66	60	61	55	63	60	71	59	57
	Method	Trans. Std.	Trans. Std.	Trans. Std.	Trans. Std.	Trans. Std.	Trans. Std.	Trans. Std.	Trans. Std.	Trans. Std.	Trans. Std.	Trans. Std.	Trans. Std.	Trans. Std.	Trans. Std.

*SD, Standard Deviation; N, Number of animals; ALT, Alanine Aminotransferase; AST, Aspartate Aminotransferase; GGT, Gamma-Glutamyl Transferase; NEFAs, Non-Esterified Fatty Acids; PigMAP, Pig Major Acute Phase Protein; TGs, Triglycerides; TNF-alpha, Tumor Necrosis Factor alpha; TP, Total Proteins*.

*Methods: NP, Non-parametric method; Trans. Std., Transformed data using standard method; Untrans. Rob., Untransformed data using robust method*.

* *Non-parametric method was used due to unfixable distribution [p-value (Anderson-darling) < 0.05 and p-value (symmetry test) < 0.01] both before and after transformation. This method was recommended by the software*.

## Discussion

A table with RI for calves and piglets has been designed for veterinary use with comparison of results in adults obtained from the literature (Tables 3, 4).

**Table 3:** Reference intervals (RIs) of clinical biochemistry analytes from calves of 24 h of age (24 h after colostrum taking) 2, 5, and 7 weeks of age and RIs of adult cattle from literature.

**Analyte**	**Unit**	**24 h**	**2 w**	**5 w**	**7 w**	**Adult**	
ALT	U/L	6.3–24.7	4.0–11.1	4.8–13.3	5.2–15.3	11–40	(2)
AST	U/L	49.6–133.4	19.5–55.0	22.0–63.3	19.0–63.3	78–132	(2)
Cholesterol	mg/dL	20.8–67.5	20.8–128.9	33.9–136.4	25.4–118.3	80–120	(2)
Creatinine	mg/dL	0.93–1.65	0.64–1.51	0.56–1.20	0.47–0.99	1.0–2.0	(2)
GGT	U/L	0.0–3201.8	16.5–262.5	12.0–48.6	11.0–27.6	6.1–17.4	(2)
Glucose	mg/dL	75.3–193.3	70.3–152.9	63.0–144.1	63.4–123.0	45–75	(2)
GPx	U/L	363.7–2116.5	270.5–1023.7	255.5–842.3	288.7–711.0	-	
Haptoglobin	mg/dL	0.09–0.31	0.07–0.36	0.07–0.46	0.08–0.38	< 1.0 ^§^	(27)
NEFAs	mmol/L	0.09–0.72	0.04–0.27	0.07–0.29	0.06–0.35	0.07–0.46	(28)
SOD	U/mL	0.05–4.57	0.04–0.84	0.05–0.62	0.03–0.57	(12.71 ± 0.34) ^*^	(29)
TGs	mg/dL	11.3–93.7	6.6–57.5	8.2–58.8	6.7–58.5	0–14	(2)
TP	g/dL	4.58–7.52	3.95–6.54	3.94–5.93	3.87–6.13	6.74–7.46	(2)
Urea	mg/dL	9.2–32.5	7.5–30.4	7.7–23.8	6.8–18.3	20–30	(2)

*ALT, Alanine Aminotransferase; AST, Aspartate Aminotransferase; GGT, Gamma-Glutamyl Transferase; GPx, Glutathione Peroxidase; NEFAs, Non-Esterified Fatty Acids; TGs, Triglycerides; TP, Total Proteins; SOD, Superoxide Dismutase*.

§ *Mean concentration obtained from 10 healthy cows*.

* *Cow in transition period (60 d before until 100 d after milk)*.

**Table 4:** Reference intervals (RIs) of clinical biochemistry analytes from piglets at 0, 7, and 14 days post-weaning (PW) (at 21, 28, and 35 days of age) and RIs of adult pig from literature.

**Analyte**	**Unit**	**0 d PW**	**7 d PW**	**14 d PW**	**Adult**	
ALT	U/L	16.5–54.2	11.4–47.1	13.6–92.7	31–58	(2)
AST	U/L	27.5–230.4	15.7–122.9	26.6–223.9	32–84	(2)
Cholesterol	mg/dL	58.9–224.3	28.7–85.5	39.8–141.2	36–54	(2)
Cortisol	ng/mL	6.9–206.5	1.4–50.8	1.0–66.7	(29.7 ± 1.0)	(2)
Creatinine	mg/dL	0.50–1.09	0.69–1.34	0.55–1.22	1.0–2.7	(2)
GGT	U/L	18.7–85.1	15.3–70.8	23.1–92.7	10–60	(2)
Glucose	mg/dL	44.0–117.8	56.4–106.3	46.6–113.6	85–150	(2)
Haptoglobin	mg/dL	0.03–1.55	0.08–2.34	0.05–0.18	0.02–3.00 ^§^	(30)
NEFAs	mmol/L	0.15–0.71	0.04–0.48	0.05–0.18	-	
PigMAP	g/L	1.33–6.14	0.51–1.73	0.36–1.39	0.46–2.36 ^§^	(30)
TGs	mg/dL	23.9–76.5	21.0–65.9	22.2–62.0	-	
TNF-alpha	pg/mL	22.0–108.4	51.9–162.4	46.3–202.6	< 10 ^*^	(31)
TP	g/dL	2.67–5.27	2.59–4.52	2.79–5.57	7.90–8.90	(2)
Urea	mg/dL	7.1–21.6	6.1–33.7	5.1–36.0	10–30	(2)

*ALT, Alanine Aminotransferase; AST, Aspartate Aminotransferase; GGT, Gamma-Glutamyl Transferase; NEFAs, Non-Esterified Fatty Acids; PigMAP, Pig Major Acute Phase Protein; TGs, Triglycerides; TNF-alpha, Tumor Necrosis Factor alpha; TP, Total Proteins*.

§ *RIs made for mixed-age pigs at 5 w old obtained from 10 commercial farms*.

* *Undetectable TNF-alpha level for 6 healthy pigs. The lower limit for detection is > 10 pg according to manufacturer*.

### RIs for Unweaned Calves

It was noticed that the all enzymes (ALT, AST, GGT, GPx, and SOD) not only showed the highest concentrations at 24 h of age, but also significantly higher than any other times of age. The high concentration in newborn animals is an indication that these enzymes are absorbed from colostrum, at least in the case of GGT and AST (9, 32). The most extreme elevation occurred with GGT, which was 20 folds higher at 24 h than at 2 w and further decreases at 5 w and 7 w. Even though the GGT level is relatively low in adult cow's serum, its level in colostrum can peak-up to ~800-fold greater than in the serum of the same cow (33). GGT can increase its activity in calves' serum as early as 6 h after colostrum intake (intake at birth) (34) and similar to our results, it has been described that it takes approximately up to 5 weeks for GGT to decline to low level (adult's level) after the intake of colostrum (3, 11, 33). Other studies found that lower concentrations of GGT in new-born calves are linked with much lower levels of globulins/total protein and higher risk of death (33, 35); moreover, one study showed 0% sensitivity of GGT when it comes to the detection of hepatic diseases before 6 w old (36) since the highly concentrated colostrum-derived GGT presented in the serum can mask the level of GGT that is derived from liver damage. Indeed, a better characterization of GGT levels for new-born calves may help to monitor calves' health to prevent early-age death but not used to detect hepatic diseases. It is also relevant to notice the high individual variability of GGT values in 24 h old calves, probably indicating differences in colostrum intake, absorption and/or metabolization.

After 24 h of age, ALT's level reached its lowest at 2 w and gradually went uphill afterwards while AST stayed relatively steady and low levels from 2 to 7 w. However, at 7 w of age, both levels of ALT and AST were still lower compared with adult's RI. Other studies with longer terms showed that AST reaches adult's level around 8–10 w of life (9, 10). According to previous study along with GGT and other enzymes (36), AST's sensitivity to liver damage is 80% for calves <6 w old thus AST alone should not be considered clinical useful in diagnosing hepatic diseases. In addition, despite the statistical differences in AST throughout 2–7 w, none of their pair-wise comparison fold changes were larger than 1.15 or <0.85, thus biologically these were not considered major differences. For ALT, its characterization in cow is seldom studied because is not so commonly used for hepatic disease diagnosis since its activity is low and isn't massively liberated into serum during hepatic diseases in large animal (2).

SOD and GPx also showed rather stable levels from 2 to 7 w. Unfortunately, we didn't find any RIs of GPx for adult; and for SOD only RI of cow during transition period (60 d before until 100 d after milk) was found. Both mineral dependent enzymes [Mn, Cu and Zn for SOD (37) and Se for (2, 38)] are important enzymes whereas SOD can dismutate two O${}_{2}^{-}$ molecules to H_2_O_2_ and O_2_ while GPx can catalyze H_2_O_2_ to H_2_O (2). Study of SOD in RBC suggested that there is no such variation in the activity between cattle and human (39). Even though there are very few information about SOD and GPx, a better characterization may contribute to the monitor of minerals and oxidation in animal's body.

NEFAs and TGs were also elevated at 24 h because colostrum is rich in fat (40); their concentrations dropped at 2 w compared to 24 h and started increasing moderately with age until 7 w. While the level of NEFAs is almost comparable with adult's RI roughly from 5 w, TG's level is still beyond the adult's RI upper limit at 7 w. TGs can richly appear in chylomicrons, which are secreted by the intestine after fat-containing meal in preruminant calves. The TGs from chylomicrons can be hydrolyzed to NEFAs and then be transported to tissue for fat storage or for oxidation to produce energy (41); on the other hand, serum NEFAs can increase in case of fasting or negative energy balance. Indeed, the growth of calf requires a large amount of energy from fat and evidences have already shown that extra fat can increase BW gains (42, 43). An adult's-like RI in NEFAs from 5 w and a rather higher levels of TGs throughout the study indicate that the calves demanded high amount of energy for growth and no negative energy balance was noticed. Another form of TGs is very-low-density lipoprotein (VLDL) but it is generally low in bovine compared with in human (41).

Cholesterol had a very similar trend with one study (9) where the lowest concentration was found at 24 h and the concentrations started to increase smoothly. Even though it was previously described that colostrum contains higher cholesterol concentration compared with milk (44) and the serum cholesterol concentration is proportional to the colostral cholesterol concentration (45), the increase in total cholesterol along with increases in NEFAs and TGs probably indicate a major lipid mobilization within tissues after 24 h.

Glucose showed significant drop throughout age. The glucose supply from mother in neonate stops abruptly immediately after birth and the suckling neonate is entirely dependent on glucose supply via colostrum/milk, meanwhile the neonate needs to rapidly turn on pathways of endogenous glucose production as well as for fatty acid oxidation to maintain its organs and cells' functionality (46). The decrease of glucose and increase of NEFAs and TGs during the first 7 w may be the consequence of the high fat and relatively low carbohydrate content of colostrum/milk (47, 48). Despite the constant fall of serum glucose throughout the study, the RIs obtained from last measurement was still higher than the adult's one.

Moreover, creatinine, TP and urea also decreased throughout age until 7 w. Creatinine is a breakdown product of creatine (Cr) and creatine phosphate (PCr) in muscle and is cleared by the kidney. In human neonatology, it was well explained that the kidneys in fetus don't play a major role in maintaining fetal homeostasis (49, 50); also, the high blood concentration of creatinine comes from maternal transfer and endogenous Cr and PCr degradation and it does not indicate fetal renal failure. It was noticed that at 2 and 5 w the median creatinine levels were around the bottom line of the adult's RI while at 7 w it was below [median and SD at 7 w was 0.66 ± 0.12 mg/dL and adult's RIs are 1.0–2.0 mg/dL according to Kaneko et al. (2) and 0.9–1.4 mg/dL according to Pérez-Santos et al. (11)], comparing to another similar study (11), where mean creatinine laid within normal adult's RI from 6 d until 90 d of age. Yet we didn't find any other issues related to the muscle or health in these calves and a possible reason of this is due to diet/season/genetic variabilities between the farms. TP was also elevated at 24 h due to passive transfer of protein which mainly contains immunoglobulins from colostrum (32, 51, 52). This result is similar to those were reported in previous studies (11, 53). Urea had a similar change pattern to the TP along the time. It is the product of protein breakdown (52) and is synthesized by the liver and finally excreted by the kidney, which is directly influenced by the total protein level.

A special attention was paid to markers of stress since assessing a good welfare status is one of the main problems in calf production. The levels of haptoglobin didn't change along the time, similar to one previous study (54) and are lying within the health adult's RI range (10 mg/dL) (27). It is important to have specific RIs for haptoglobin in young calves since it is an acute-phase protein used as biomarker of inflammatory and infectious diseases (55), very common in young calves. The individuals with high haptoglobin values identified as outliers may be individuals with subclinical diseases, which were recognized as healthy in a clinical inspection. The antioxidant enzymes GPx and SOD, markers of oxidative stress (56) had higher values at 24 h, but the RIs are similar afterwards.

As it was previously mentioned, on week 2 we found trial-biased distributions (Figure S1) on AST and cholesterol, where T3 showed lower concentration range compared with others. This reason may be because the average age in T3 is ~2 d older than other two. This difference indicates that only 2-days age difference can cause mean concentration difference in some parameters, thus special care should be taken when comparing local clinical values with RIs in literature.

### RIs for Post-weaning Piglets

No major changes were noticed on hepatic enzymes (ALT, AST, and GGT), glucose and TP. Cholesterol, NEFAs and TGs showed their highest values just after weaning (0 d). This may be due to the sudden dietary change from high fat content of milk to less-digestible, more-complex solid feed that piglets eat after weaning (57).

Throughout the ages, all medians of urea were within adult's RI while all medians of creatinine were <0.8 folds to the adult's RI lower fence. Both analytes showed highest concentrations at 7 d post-weaning. The increase in urea and creatinine only at 7 d post-weaning may be due to transient dehydration after the dietary change. These pattern changes are very similar to one study between 5-day-old unweaned piglets and 30-day-old post-weaning piglets (weaned at 28 d of life) supplemented with iron in solid diet (12). However, since this report only measured 5 d and 30 d, different from our opinion, the author's explanation of the increases was due to the increase of muscle mass and helped by the gradual supplementation of enriched solid diet.

Special interest has been devoted to markers of stress and inflammation. Thus, cortisol was measured as the paradigmatic stress hormone and found higher just after weaning, similar to other studies (58–60), this probably is due to the stress associated to separation of the mother and change of diet; it's also interesting to notice that from 7 d post-weaning, the mean concentrations of piglets are comparable to healthy adults' one (31), which suggests that in this study the diet change only caused a transient stress to the animals. The acute phase proteins haptoglobin and Pig-MAP were also measured; whereas haptoglobin RIs were not influenced by age and weaning, Pig-MAP was higher at 3 weeks-old just after weaning. Absolute values for haptoglobin were similar to those reported at 3-week-old (13). In that study, reference values for both acute phase proteins were established for pigs older than 4 weeks. Pig-MAP is also considered a stress marker, which increases in stressful conditions like transport (61–63). Finally, the cytokine TNF-alpha is frequently used as a marker of infection, especially in gastrointestinal disorders which often affect piglets at weaning (31, 64, 65). For this reason, it is interesting to have RIs available in healthy animals. A moderate increase in TNF-alpha was shown at 7 and 14 days after weaning, probably due to the change of diet. This increase in serum may be secondary to the transient response in gene expression of inflammatory cytokines in the gut after weaning (66).

Similar to the calves, creatinine from 0 d post-weaning (3 w of age) was also comparable to the adult's RIs. GGT's RI is also comparable to adult's one from 7 d of age while for calves it takes 5 weeks to drop into adult's RI (2).

## Conclusions

Specific information about reference intervals for young animals is a requirement for the adequate clinical and nutritional management of the individuals. Even in farm animals, where collective evaluation of health is frequent instead of an individual assessment, the availability of specific RIs for young ages is essential to for an accurate diagnosis of health problems. In this sense, most important life periods in these two farm animal species: in cattle, colostrum uptake and suckling is a critical period during which the calf starts growing and obtains the immune defenses necessary for a sustained growth. In piglets, the post-weaning period is the most critical due to the high incidence of gastrointestinal and respiratory disorders. In this work, we have used a large number of individuals to obtain RIs specific for calves and piglets at these critical stages, and the most relevant parameters related to these stages evaluated.

The first set of analytical parameters are related to nutrition and metabolism, i.e., indicators of carbohydrate, lipid, and protein metabolism. Many health problems in young animals arise from nutritional unbalances and management procedures.

Secondly, enzymatic markers of oxidative stress were also evaluated. Oxidative stress is a relevant issue in cattle (67) and, specifically, neonates are particularly susceptible because of the challenge due to the transition from the hypoxic intrauterine environment to extrauterine life, susceptibility of neonates to infection, and limited antioxidant protection (68). In piglets, several diseases with severe consequences on health occur concomitantly with oxidative stress (56).

Third, markers of stress (cortisol) and inflammation/infection have been also evaluated. In this sense, the TNF-alpha cytokine and the acute phase proteins Pig-MAP and haptoglobin have been analyzed for piglets and calves, respectively.

This information will help veterinarians and animal science researchers to better practice their professions by providing them with more adequate diagnostic tools.

## Ethics Statement

Male Holstein calves were managed according to the recommendations of the Animal Care Committee of Institut de Recerca i Tecnologia Agroalimentàries (IRTA) under the approval research protocol FUE-2017-00587321, authorization code 9733. Piglets' procedure has been approved by ethical committee (CEEAH) of Universitat Autònoma de Barcelona (Registration no: 1406 of the Departament de Medi Ambient i Habitatge of the Generalitat de Catalunya).

## Author Contributions

KY performed the manuscript writing, RI establishment and statistics analyses. FC provided statistical advices. Handling of animals and sample collection was performed by DS-O for piglets and MT for calves. LA, RP, and YS performed sample analyses and AB conducted in the conceptualization, supervised the analytical work, and revised the manuscript. All authors read and approved the final manuscript.

### Conflict of Interest Statement

The authors declare that the research was conducted in the absence of any commercial or financial relationships that could be construed as a potential conflict of interest.
